# Mitochondrial Respiration in Peripheral Blood Cells Links to Metabolic Flexibility and Physical Performance in Ageing

**DOI:** 10.1002/jcsm.70361

**Published:** 2026-09-02

**Authors:** Donna Li, Catrin Herpich, Christopher Bishop, Marie Aleithe, Antonia Peil, Nele Krüger, Jannes Felsner, Lea Göger, Maximilian Kleinert, Kristina Norman

**Affiliations:** ^1^ Institute of Nutritional Sciences University of Potsdam Nuthetal Germany; ^2^ Department of Nutrition and Gerontology German Institute of Human Nutrition Potsdam‐Rehbruecke Nuthetal Germany; ^3^ Department of Molecular Physiology of Exercise and Nutrition German Institute of Human Nutrition (DifE) Potsdam‐Rehbruecke Nuthetal Germany; ^4^ German Center for Diabetes Research (DZD) Munich Germany; ^5^ Department of Geriatrics and Medical Gerontology Charité—Universitätsmedizin Berlin, Corporate Member of Freie Universität Berlin and Humboldt‐Universität zu Berlin Berlin Germany; ^6^ German Center for Cardiovascular Research (DZHK), Partner Site Berlin Berlin Germany

**Keywords:** ageing, metabolic flexibility, metabolism, mitochondrial respiratory capacity

## Abstract

**Background:**

Age‐related declines in energy metabolism, muscle strength and physical performance have been linked to lower mitochondrial respiratory capacity. Peripheral blood mononuclear cell (PBMC) respiration offers a minimally invasive marker of systemic bioenergetics, yet its relationship to whole‐body metabolic flexibility remains unclear. This study examined whether PBMC respiratory capacity is associated with substrate utilization during submaximal exercise, muscle strength and physical function in healthy older adults.

**Methods:**

PBMC mitochondrial respiratory capacity was quantified by high‐resolution respirometry assessing ROUTINE, LEAK and MAX states. Postprandial substrate oxidation during steady‐state treadmill walking at 60% of VO_2_max (oxygen uptake) was quantified by indirect calorimetry, and fat and carbohydrate oxidation rates were calculated using standard stoichiometric equations. Metabolic flexibility was defined as lower respiratory exchange ratio (RER) and higher relative fat oxidation at a fixed workload. Muscle strength was determined by handgrip dynamometry and one‐repetition maximum leg extension. Physical function was evaluated by gait speed and five‐repetition chair rise time. Associations were tested with linear and logistic regression adjusted for age, sex, skeletal muscle index, physical activity and high‐sensitive C‐reactive protein concentrations. Exploratory K‐means clustering identified mitochondrial respiration phenotypes.

**Results:**

Fifty community‐dwelling older adults (22 men, 28 women; age 70 ± 4 years) were examined. Higher ROUTINE respiration was correlated with RER (rho = −0.335, *p* = 0.020), fat utilization (rho = −0.334, *p* = 0.019), grip strength (rho = 0.302, *p* = 0.033) and gait speed (rho = 0.324, *p* = 0.022). Adjusted regression analyses confirmed the association of ROUTINE respiration with greater fat oxidation (β = 0.212, 95% CI 0.049; 0.375), lower RER (β = −0.160, 95% CI −0.300; −0.020) and higher gait speed (β = 0.153, 95% CI 0.028; 0.277). Similar associations were found for ATP‐linked respiration. Cluster analysis identified high‐ and low‐respiration phenotypes. Compared with the high‐respiration group the low‐respiration group showed lower CMJ height (OR: 0.204, 95% CI 0.055; 0.763) and quadriceps strength (OR: 0.373, 95% CI 0.155; 0.897).

**Conclusions:**

In healthy older adults, higher PBMC ROUTINE respiration was associated with a more fat‐dominant substrate utilization profile during submaximal exercise, greater muscle strength and faster gait speed. PBMC respiratory capacity may reflect systemic bioenergetic status relevant to exercise substrate utilization and physical performance in ageing.

## 1 Introduction

Ageing is associated with a range of physiological declines that impact cardiovascular, metabolic and musculoskeletal systems. These include declines in cardiorespiratory fitness, efficiency of energy utilization and reductions in muscular strength and functional performance. Collectively, such changes contribute to metabolic burden and disease, as well as reduced functional capacity, greater risk of falls and overall declines in quality of life [1, 2].

Mitochondria play a central role in maintaining systemic health and their altered respiratory capacity is recognized as a hallmark of ageing. This alteration of mitochondrial respiratory capacity is characterized in part by reduced bioenergetic capacity and oxidative efficiency and is linked to impaired cellular energy metabolism in age‐related conditions [3]. These declines, seen across multiple tissues, including skeletal muscle, brain and immune cells, may impair the body's ability to switch between energy substrates, a capacity known as metabolic flexibility [4].

Metabolic flexibility refers to the capacity to shift between fat and carbohydrate oxidation in response to metabolic demands, such as transitioning from rest to moderate or vigorous exercise. At rest or lower intensities up to 65% of VO_2_max (the maximum rate of oxygen consumption during exercise), fat is typically the predominant fuel, whereas carbohydrate use increases with greater intensity [5]. This adaptive switching appears to be blunted with ageing [6] and conditions such as sarcopenia [7] and may reflect impairments in the overarching function of the mitochondria.

Mitochondrial respiratory capacity has been linked to VO_2_max, muscular strength, insulin sensitivity and overall physical performance in older adults, highlighting its integrative role in whole‐body systems [8, 9, 10]. One minimally invasive and accessible way to capture a snapshot of systemic mitochondrial health is through mitochondrial assessment in peripheral blood mononuclear cells (PBMCs) [11].

Previous work, including by our group, has shown that PBMC mitochondrial respiratory capacity is altered in age‐related fatigue [12] and several disease conditions such as Type 2 diabetes [13], cardiovascular disease [14] and obesity [15]. Other work has shown an association with physical function where higher maximal PBMC respiration was associated with better physical function markers such as gait speed and lower‐extremity strength in community‐dwelling and overweight or obese older adults [15, 16]. What remains unclear is whether PBMC mitochondrial function is related to metabolic flexibility, particularly given its proposed role in systemic energy regulation and substrate utilization.

Therefore, the purpose of this study was to investigate whether mitochondrial respiration parameters measured in PBMCs are associated with metabolic flexibility and physical function in healthy older adults. Metabolic flexibility was assessed through steady‐state substrate utilization during submaximal exercise, whereas physical function was evaluated using various measures of muscle strength and functional performance. We hypothesized that greater mitochondrial efficiency, reflected by higher routine and maximal respiration, is related to greater fat oxidation and lower respiratory exchange ratio (RER) during submaximal exercise, as well as better strength and functional performance. In exploratory analyses, we also used unsupervised clustering to identify distinct mitochondrial phenotypes and evaluated whether these groups differed in metabolic and physical performance.

## 2 Methods

This was a cross‐sectional observational study conducted at the German Institute of Human Nutrition Potsdam‐Rehbruecke.

### 2.1 Participants

Of the 133 community‐dwelling older adults assessed for eligibility, 50 (22 men, 28 women) were included in this analysis. Participants were recruited through community advertisements between December 2023 to March 2025. Inclusion criteria were: aged between 65 and 85 years, recreationally active but not engaging in structured aerobic or resistance exercise more than twice weekly, body mass index (BMI) between 22 and 30 kg/m^**2**^, and non‐smokers. Exclusion criteria included food allergies or intolerances to seafood/algae, blood pressure exceeding 140/90 mmHg, atrial fibrillation, the use of blood thinners or self‐reported history of severe or active liver, kidney, rheumatic or malignant disease. No formal sample size calculation was conducted for this study; the number of participants reflects recruitment feasibility. All participants gave written informed consent in accordance with the Declaration of Helsinki. The study protocol was approved by the Ethics Committee of the University of Potsdam and registered (DRKS00032985).

### 2.2 Anthropometry and Body Composition

Participants arrived early in the morning after an overnight fast. Weight and height were measured and used to calculate BMI. Body composition was assessed using an octopolar multi‐frequency BIA device (seca mBCA 525, Germany). Outputs included fat mass and skeletal muscle mass, which were used to derive skeletal muscle index (SMI) and fat mass index (FMI), both calculated as divided by height squared (kg/m^**2**^). Habitual physical activity level (low, moderate and high) was evaluated using the International Physical Activity Questionnaire short form [17].

### 2.3 PBMC Mitochondrial Respiration and Inflammation

Venous blood was collected in EDTA tubes and processed within 3 h. After diluting the blood 1:1 with phosphate buffered saline (PBS), PBMCs were isolated using SepMate tubes with Lymphoprep density gradient centrifugation (1200 g, 10 min). Cells were washed twice with PBS, centrifuged (300 g, 10 min) and resuspended in PBS. Viable cells were counted using trypan blue staining in a Neubauer chamber. The 4 × 10^6^ viable cells in 2.3 mL were needed to conduct respirometry.

Mitochondrial respiration was assessed in 4 × 10^6^ freshly isolated PBMCs using high‐resolution respirometry (Oxygraph‐O2k, OROBOROS Instruments, Innsbruck, Austria) at 37°C in 2.3 mL of MiR05 respiration buffer, with continuous stirring. ROUTINE respiration (basal oxygen consumption reflecting physiological adenosine triphosphate [ATP] turnover) was measured first, followed by LEAK respiration (non‐phosphorylating oxygen consumption after ATP synthase inhibition with oligomycin A, 2.5 μM) and MAX respiration (uncoupled maximal capacity, induced by titration of FCCP, 0.5–2 μM). Finally, non‐mitochondrial oxygen consumption (ROX) was determined after inhibition with rotenone (0.5 μM) and antimycin A (2.5 μM). All oxygen fluxes were corrected for ROX and expressed relative to cell count (pmol O_2_·s^−**1**^·10^6^ cells^−1^). The difference of MAX and ROUTINE respiration was calculated as reserve capacity, whereas the difference of ROUTINE and LEAK represented ATP‐linked respiration. As an inflammatory marker, high‐sensitive C‐reactive protein (CRP) was quantified in blood serum using ELISA (apDia, 74001, Turnhout, Belgium).

### 2.4 Muscle Strength Testing

#### 2.4.1 Leg Extension One‐Repetition Maximum (1RM)

Quadriceps strength was determined via a one‐repetition maximum (1RM) test on a leg extension machine (MAXXUS, Germany). Participants performed a warm‐up of eight repetitions at ~30% estimated 1RM, followed by progressive trials of increasing load and decreasing repetitions. Rest periods of 2 min were taken between attempts. Encouragement was provided during each attempt. The 1RM was defined as the maximum load lifted once with correct form. The test ended after two failed attempts at a given weight as determined by trained study personnel. The value was normalized to body weight (kg/kg) in further analysis.

#### 2.4.2 Grip Strength

Grip strength was assessed using a handgrip dynamometer (Leonardo Mechanograph GF, Novotec Medical, Germany). Participants were seated with their elbow flexed at 90°, forearm in a neutral position. They were instructed to squeeze the device as forcefully as possible for approximately 5 s. Three trials were performed with the dominant hand, separated by 60 s of rest, and the highest value (kg) was used for analysis. Grip strength was normalized to body weight (kg/kg) for further analysis.

### 2.5 Muscle Function Testing

#### 2.5.1 Chair Rise Test

Lower‐limb functional performance was evaluated using the five‐repetition chair rise test conducted on a force plate (Leonardo Mechanograph GF, Novotec Medical, Germany). Participants began seated on a standardized bench (43 cm height) with arms crossed over the chest. They were instructed to rise to a full stand and return to seated position five times as quickly as possible. Timing began on the first upward motion and was stopped when the participant returned to the seated position after the fifth rise. The best time of two trials was recorded (s).

#### 2.5.2 Gait Speed

Gait speed was assessed using a 4‐m walk test. Participants were instructed to walk at their usual pace along between two marked lines. Timing began when the participant's first foot crossed the starting line and ended when the first foot crossed the 4‐m finish line. The test was performed twice, and the fastest time was used to calculate gait speed (m/s).

#### 2.5.3 Countermovement Jump (CMJ)

Countermovement jump (CMJ) was performed on a force measurement platform (Leonardo Mechanograph GF). Participants stood with feet shoulder‐width apart, hands on hips to restrict arm swing, and performed maximal vertical jumps. Two trials were performed with a 60‐s rest in between. The highest jump was used for analysis (cm).

### 2.6 Submaximal Exercise

Substrate utilization was assessed during a 10‐min treadmill walking protocol at 60% of estimated VO_2_max. This steady‐state submaximal protocol was selected to provide a feasible and standardized assessment of exercise substrate utilization in older adults. To reduce variability, participants completed testing under standardized postprandial conditions, prior movement was minimized, and exercise testing was performed under similar laboratory timing across participants. Target intensities were derived using the SHIP [18] and FRIEND [19] equations, as there is no established standardized equation for estimating VO_2_max in older adults. Participants were allotted 15 min to consume a standardized test meal (345 kcal: 56 g carbohydrates, 9.2 g fat, 6.9 g protein) prior to testing. Approximately 10 min following the cessation of the meal, a 10‐min warm‐up at a subjectively comfortable walking pace was performed prior to the exercise bout to familiarize individuals to the treadmill. Once the target workload was reached, the 10‐min breath‐by‐breath gas exchange data were collected using a calibrated metabolic system (MetaMax 3B, Cortex Biophysik, Leipzig, Germany). RER was calculated as the ratio of carbon dioxide production (VCO_2_) to oxygen consumption (VO_2_), averaged over the final 2 min of the steady‐state phase. Fat and carbohydrate oxidation rates were derived from VO_2_ and VCO_2_ values using the non‐protein stoichiometric equations described by Frayn [20]. To express relative substrate utilization, these oxidation rates were expressed as percentage contribution to total energy expenditure during steady‐state exercise.

### 2.7 Metabolic Flexibility

Metabolic flexibility was operationally defined as substrate preference during exercise. For instance, lower RER or higher fat oxidation/utilization was interpreted as greater flexibility, and higher RER or higher carbohydrate oxidation/utilization was considered as reduced flexibility.

### 2.8 Statistical Analysis

Statistical analyses were performed using IBM SPSS Statistics (Version 28) and Python (3.13.0). Missing data were less than 5% for all variables and were handled with complete‐case analysis. Spearman's rank correlation was used to evaluate associations between PBMC respiration and performance variables. Linear and logistic regressions assessed relationships between mitochondrial respiration and outcomes related to metabolic flexibility and physical performance. Multiple linear regressions, adjusting for age, sex, SMI, physical activity level and hsCRP concentrations, were performed on log‐transformed values of ROUTINE respiration and cluster membership. All variables were z‐score standardized. K‐means clustering based on log‐transformed ROUTINE, LEAK and MAX respiration values was performed to identify distinct mitochondrial phenotypes. Significance was defined as *p* < 0.05 or when the 95% CI of the regression coefficient or odds ratio did not cross zero or one respectively. Visualizations were created with python packages, matplotlib, seaborn and scipy.

## 3 Results

### 3.1 Participant Characteristics

Fifty community‐dwelling older adults (22 men, 28 women) were analysed. Table 1 displays baseline characteristics where, as expected, men had lower FMI but higher SMI compared to women. They also demonstrated greater grip strength, jump height performance and fat/carbohydrates oxidation, although no significant sex differences were observed in mitochondrial respiration parameters nor other functional parameters (CRT and gait speed).

**TABLE 1:** Baseline characteristics of the study population.

Variable	Men (*n* = 22)	Women (*n* = 28)	*p*
Age (years)	71.06 ± 4.59	69.88 ± 4.80	0.269
Medication (number)	1 ± 1	1 ± 1	0.742
hsCRP (mg·L^−1^)	1.54 ± 2.40	2.32 ± 3.98	0.388
BMI (kg/m^2^)	25.49 ± 2.38	24.54 ± 3.18	0.369
FMI (kg/m^2^)	6.86 ± 1.90	9.06 ± 2.79	0.010
SMI (kg/m^2^)	8.82 ± 0.81	6.85 ± 0.68	< 0.001*
ROUTINE (pmol O_2_·s^−1^·10^6^ cells^−1^)	3.11 ± 1.10	2.86 ± 1.31	0.348
LEAK (pmol O_2_·s^−1^·10^6^ cells^−1^)	0.94 ± 0.47	1.07 ± 0.59	0.470
MAX (pmol O_2_·s^−1^·10^6^ cells^−1^)	6.92 ± 2.35	6.56 ± 2.98	0.348
Reserve capacity (pmol O_2_·s^−1^·10^6^ cells^−1^)	3.80 ± 1.80	3.69 ± 2.42	0.379
ATP‐linked respiration (pmol O_2_·s^−1^·10^6^ cells^−1^)	2.17 ± 0.89	1.80 ± 0.95	0.184
RER	0.89 ± 0.02	0.88 ± 0.03	0.258
Fat oxidation (g/min)	0.25 ± 0.06	0.17 ± 0.05	< 0.001*
CHO oxidation (g/min)	1.08 ± 0.20	0.63 ± 0.12	< 0.001*
Fat utilization (%)	36.34 ± 8.32	39.31 ± 10.45	0.191
CHO utilization (%)	63.66 ± 8.32	60.69 ± 10.45	0.191
1RM/BW (kg/kg)	1.29 ± 0.19	1.19 ± 0.37	0.278
Grip Strength/BW (kg/kg)	0.50 ± 0.06	0.39 ± 0.08	< 0.001*
CMJ height (cm)	27.18 ± 5.44	18.31 ± 4.02	< 0.001*
Gait speed (m/s)	1.35 ± 0.16	1.34 ± 0.23	0.814
CRT time (s)	9.28 ± 1.87	9.80 ± 2.14	0.394

Abbreviations: 1RM/BW = one‐repetition maximum normalized to body weight, BMI = body mass index, CHO = carbohydrate, CMJ = countermovement jump, CRT = chair rise test, FMI = fat mass index, Handgrip/BW = handgrip strength normalized to body weight, LEAK = mitochondrial proton leak respiration, MAX = maximal mitochondrial respiration, RER = respiratory exchange ratio, ROUTINE = routine mitochondrial respiration, SMI = skeletal muscle mass index.

* Statistical significance based on the adjusted *p*‐value of 0.002.

#### 3.1.1 Mitochondrial Bioenergetics and Metabolic Flexibility

**FIGURE 1:**
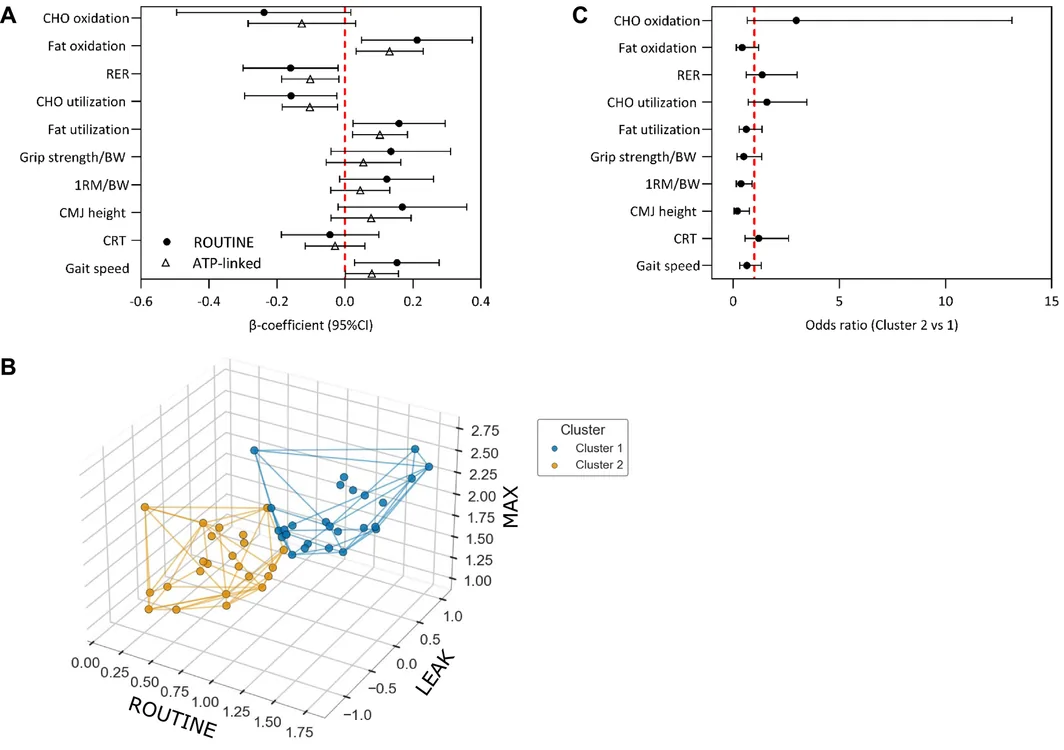
(A) Forest plot of associations between ROUTINE and ATP‐linked mitochondrial respiratory capacity and metabolic flexibility as well as performance outcomes. (B) K‐means cluster analysis of mitochondrial respiration reveals two distinct bioenergetic profiles. Beta coefficients (β) and 95% confidence intervals (CI) are displayed for each outcome. Associations were considered statistically significant when the 95% CI did not cross zero. (C) Forest plot of associations between cluster membership and metabolic flexibility as well as performance outcomes. Odds ratios with 95% CI, adjusted for age, sex, SMI, physical activity level and hsCRP. No units are shown as variables were z‐standardized. Associations were considered statistically significant when the 95% CI did not cross one. 1RM/BW = one‐repetition maximum normalized to body weight, CHO = carbohydrate, CMJ = countermovement jump, CRT = chair rise test, Grip Strength/BW = handgrip strength normalized to body weight, RER = respiratory exchange ratio, SMI = skeletal muscle index.

Spearman correlation analyses revealed ROUTINE mitochondrial respiration was negatively correlated with RER and carbohydrate utilization as well as positively correlated with fat utilization (Table 2), which was also found for ATP‐linked respiration. As LEAK nor MAX were significantly correlated with metabolic flexibility markers, linear regression analysis was only performed on ROUTINE and ATP‐linked measures. The same associations were upheld in adjusted linear regression models (Figure 1A).

**TABLE 2:** Correlations between PBMC respiration parameters and metabolic and physical function outcomes.

Outcome measure	ROUTINE		LEAK		ATP‐linked		MAX	
	rho	*p*	rho	*p*	rho	*p*	rho	*p*
RER	−0.335	0.020	−0.118	0.426	−0.356	0.013	−0.211	0.151
Fat oxidation	0.253	0.080	−0.091	0.533	0.327	0.022	0.219	0.130
CHO oxidation	−0.054	0.710	−0.112	0.445	−0.025	0.864	0.082	0.578
Fat utilization	0.334	0.019	0.126	0.389	0.341	0.017	0.173	0.235
CHO utilization	−0.334	0.019	−0.126	0.389	−0.341	0.017	−0.173	0.235
Grip Strength/BW	0.302	0.033	0.139	0.335	0.225	0.117	0.193	0.180
1RM/BW	0.152	0.297	0.343	0.016	−0.005	0.972	0.006	0.966
CMJ height	0.243	0.097	0.042	0.778	0.227	0.120	0.239	0.101
Gait speed	0.324	0.022	0.240	0.093	0.293	0.039	0.124	0.392
CRT time	0.037	0.803	0.101	0.493	−0.110	0.464	−0.106	0.471

*Note:* No units are shown as variables were z‐standardized. Mitochondrial respiration parameters were log‐transformed.

Abbreviations: 1RM/BW = one‐repetition maximum normalized to body weight, CHO = carbohydrate, CMJ = countermovement jump, CRT = chair rise test, Grip Strength/BW = handgrip strength normalized to body weight, RER = respiratory exchange ratio.

#### 3.1.2 Physical Function and Strength

ROUTINE and ATP‐linked respiration were significantly associated with indicators of physical performance. Specifically, ROUTINE showed a positive correlation with grip strength and gait speed (Table 2). ATP‐linked respiration correlated positively with gait speed as well. The relationship with gait speed remained true in the adjusted regression model, reinforcing the robustness of this association (Figure 1A).

In contrast, LEAK respiration was positively associated with 1RM quadriceps strength, indicating that proton leak‐related respiration may reflect lower‐body muscular strength (Table 2).

### 3.2 Cluster Analysis

To explore whether distinct mitochondrial bioenergetic phenotypes exist, K‐means clustering based on normalized ROUTINE, LEAK and MAX respiration was performed. Two clusters were identified (Figure 1B). Participants in Cluster 1 (high respiration) compared to Cluster 2 (low respiration) showed significantly higher ROUTINE respiration (3.72 ± 1.11 vs. 2.10 ± 0.60, *p* < 0.001), higher LEAK respiration (1.35 ± 0.48 vs. 0.61 ± 0.25, *p* < 0.001) and higher MAX respiration (7.78 ± 2.82 vs. 5.46 ± 1.96, *p* < 0.001).

Then, group differences were assessed to determine whether cluster membership was reflected in measures of both metabolic flexibility and strength and function variables. Indeed, significant group differences emerged in measures of metabolic flexibility. Cluster 1 or those with a higher respiration profile had significantly lower RER during submaximal exercise (0.88 ± 0.02 vs. 0.89 ± 0.03, *p* = 0.047) and higher fat utilization (39.7 ± 7.88% vs. 35.8 ± 11.1%, *p* = 0.026), suggesting greater metabolic flexibility.

Strength and function variables did not differ significantly between groups. However, there was a trend for higher 1RM quadriceps strength in Cluster 1 (1.31 ± 0.28 kg/kg vs. 1.14 ± 0.32 kg/kg, *p* = 0.067), indicating a possible relationship between mitochondrial phenotype and lower‐limb strength. In addition, participants in Cluster 1 also exhibited higher hsCRP concentrations (2.81 ± 4.33 μg/mL vs. 1.01 ± 1.12 μg/mL, *p* = 0.044).

Furthering the examination of these group differences, adjusted logistic regressions revealed that higher CMJ height and 1RM quadriceps strength were associated lower odds for Cluster 2 (low respiration) (Figure 1C).

## 4 Discussion

The concurrent decline in mitochondrial respiratory capacity, physical performance and cardiovascular capacity with age suggests a shared physiological underpinning that builds upon mitochondrial dysfunction as a hallmark of ageing. This analysis explored these connections by assessing PBMC mitochondrial respiration and its relation to postprandial substrate utilization and physical performance in healthy older adults. Several key findings yielded from the analysis: (1) Higher ROUTINE and ATP‐linked mitochondrial respiration were linked to substrate utilization, as defined by lower RER and higher fat use and oxidation during submaximal exercise. (2) ROUTINE respiration also showed positive associations with strength and functional outcomes. (3) LEAK respiration was related to leg extension strength. (4) Exploratory K‐means clustering revealed two mitochondrial phenotypes, a high and a low respiration group, with the low function group showing weaker lower body strength.

ROUTINE and ATP‐linked respiration were negatively correlated with RER, indicating that higher mitochondrial respiratory capacity and efficient oxidative phosphorylation were associated with a more fat‐dominant metabolic profile during moderate‐intensity exercise. This suggests greater metabolic flexibility as the individual is capable of holding onto a greater proportion of fat oxidation before making the switch to carbohydrates. Generally, fat oxidation contributes significantly to total energy expenditure during low‐ to moderate‐intensity exercise and reaches a peak at an intensity of 44%–65% of VO_2_max before declining as intensity increases [5, 21]. This peak, termed maximal fat oxidation (MFO), has been negatively correlated with RER during exercise at 60% VO_2_max [22]. Higher fat oxidation at 60% VO_2_max, as seen in our high‐ROUTINE individuals, may reflect a higher MFO. MFO has also been associated with higher cardiorespiratory fitness [23] and improved insulin sensitivity [21]. In our study, ROUTINE respiration and ATP‐linked respiration were also positively associated with fat utilization and negatively associated with carbohydrate utilization, further supporting potential enhanced metabolic flexibility. ROUTINE and ATP‐linked respiration also remained significantly associated with RER after adjusting for age, sex, SMI, hsCRP and physical activity level reinforcing the link between mitochondrial respiratory capacity and the exercising metabolic profile. Overall, these findings suggest that higher ROUTINE and ATP‐linked respiration may indicate greater metabolic flexibility during moderate‐intensity exercise. Because all participants completed the exercise test after the same standardized meal and meal‐to‐exercise timing, interindividual variability in nutritional state was reduced; however, these findings reflect substrate utilization during a single fixed‐intensity submaximal exercise bout and therefore represent one aspect of metabolic flexibility.

In contrast, LEAK and MAX respiration did not show significant associations with RER nor substrate utilization. This may suggest that basal mitochondrial activity (ROUTINE), which reflects endogenous ATP turnover, is more closely aligned with whole‐body substrate metabolism during submaximal exercise than stress‐induced respiration states such as LEAK or MAX, which represent non‐ATP‐linked proton leak and uncoupled maximal capacity, respectively [24]. MAX respiration was not associated with any of the parameters. This may be intrinsically due to the submaximal nature of the exercise because MAX respiration reflects the theoretical upper limit, a limit that is likely not reached under typical physiological conditions [25].

In consideration of performance metrics, ROUTINE respiration was significantly associated with upper and lower body strength as well as the functional measure gait speed. Also, higher LEAK was associated with greater 1RM quadriceps strength. These results are consistent with prior work by Tyrrell et al., who reported that higher PBMC respiration, particularly maximal and spare respiratory capacity, was associated with grip strength, extended short physical performance battery scores, knee extensor strength and leg muscle quality in overweight and obese older adults [15, 16]. In contrast, the present study examined ROUTINE respiration in a healthier, community‐dwelling population, where the association between unstimulated mitochondrial activity and physical performance suggests that even non‐stress‐induced respiration may reflect underlying differences in functional capacity. These findings extend the relevance of PBMC‐derived mitochondrial metrics beyond high‐risk cohorts. The observed associations likely reflect the integrative role of mitochondrial activity in both muscle energetics and broader physiological systems such as energy metabolism and neuromuscular coordination [11].

K‐means clustering based on ROUTINE, LEAK and MAX respiration identified two mitochondrial phenotypes. Individuals in the high‐respiration cluster (Cluster 1) demonstrated greater metabolic flexibility, as reflected by significantly lower RER and higher fat utilization during submaximal exercise, indicating more efficient substrate allocation. These findings were further supported by multiple linear regressions, which showed significantly lower 1RM quadriceps strength in Cluster 2 compared to Cluster 1. This pattern parallels observations from the Baltimore Longitudinal Study of Aging, where higher mitochondrial respiration clustered with skeletal muscle oxidative capacity and in vivo ^31^P‐MRS markers of mitochondrial health [4]. It seems that those with better substrate utilization during exercise also display greater strength, although with no differences in functional performance. However, this could simply be due to the overall healthier nature of the study participants.

Taken together, our findings demonstrate that PBMC mitochondrial respiration is associated with both metabolic flexibility and physical performance markers in healthy older adults.

## 5 Limitations

Several limitations should be acknowledged. The cross‐sectional design limits causal interpretations between mitochondrial respiratory capacity and physical or metabolic outcomes. Even though normalization to viable cell count was employed, inherent heterogeneity within bioenergetic profiles of PBMC subtypes (monocytes and lymphocytes) could contribute to observed differences in basal respiration. Systemic inflammation might affect overall respiration profiles due to an increased proportion of monocytes, which we were not able to quantify. Indeed, participants in the high respiration cluster exhibited higher hsCRP compared to participants with low respiratory capacity. Therefore, it is possible that subset‐specific inflammatory pathways may have played a role, and we have accounted for that by adjusting the regression analyses for hsCRP concentrations. Although PBMCs provide a minimally invasive means to assess mitochondrial bioenergetics, they may not fully capture the tissue‐specific respiratory capacity of mitochondria in skeletal muscle. Accordingly, the present study captures postprandial substrate utilization during a steady‐state exercise bout rather than the dynamic range of metabolic flexibility, which provided a feasible and standardized assessment in older adults. Nevertheless, to disentangle nutritional effects fasted versus fed comparisons are warranted. Lastly, the study sample consisted of healthy, recreationally active older adults, which reduces generalizability to metabolically compromised populations or older adults with frailty.

## 6 Conclusion

This study demonstrates that mitochondrial respiratory capacity in PBMCs is associated with substrate utilization during a standardized postprandial submaximal exercise in healthy older adults. Furthermore, higher respiratory capacity was linked to better handgrip strength, faster gait speed and greater lower‐limb strength. Clustering further revealed a high‐respiration phenotype with a more favourable metabolic profile and preservation of quadriceps strength and function. These findings contribute to how mitochondrial bioenergetics in immune cells relate to whole‐body metabolic responses and physical capacity in older adults.

## Funding

The authors have nothing to report.

## Ethics Statement

The study protocol was approved by the Ethics Committee of the University of Potsdam (approval number DRKS00032985). All procedures were conducted in accordance with the ethical standards of the 1964 Declaration of Helsinki and its later amendments. Written informed consent was obtained from all participants prior to inclusion in the study.

## Conflicts of Interest

The authors declare no conflicts of interest.
